# Silica-sol-based spin-coating barrier layer against phosphorous diffusion for crystalline silicon solar cells

**DOI:** 10.1186/1556-276X-9-659

**Published:** 2014-12-05

**Authors:** Abdullah Uzum, Ken Fukatsu, Hiroyuki Kanda, Yutaka Kimura, Kenji Tanimoto, Seiya Yoshinaga, Yunjian Jiang, Yasuaki Ishikawa, Yukiharu Uraoka, Seigo Ito

**Affiliations:** 1 Department of Electrical Engineering and Computer Sciences, Graduate School of Engineering, University of Hyogo, 2167 Shosha, Himeji, Hyogo 671-2280, Japan; 2 Specialty Materials Research Laboratory, Nissan Chemical Industries, Ltd., 11-1 Kitasode, Sodegaurashi, Chiba 299-0266, Japan; 3 Graduate School of Materials Science Nara Institute of Science and Technology, 8916-5, Takayama, Ikoma, Nara 630-0192, Japan

**Keywords:** CZ-Si, Spin-coating, Phosphorus barrier, Sol-gel, Silica nanoparticle

## Abstract

The phosphorus barrier layers at the doping procedure of silicon wafers were fabricated using a spin-coating method with a mixture of silica-sol and tetramethylammonium hydroxide, which can be formed at the rear surface prior to the front phosphorus spin-on-demand (SOD) diffusion and directly annealed simultaneously with the front phosphorus layer. The optimization of coating thickness was obtained by changing the applied spin-coating speed; from 2,000 to 8,000 rpm. The CZ-Si p-type silicon solar cells were fabricated with/without using the rear silica-sol layer after taking the sheet resistance measurements, SIMS analysis, and SEM measurements of the silica-sol material evaluations into consideration. For the fabrication of solar cells, a spin-coating phosphorus source was used to form the n^+^ emitter and was then diffused at 930°C for 35 min. The out-gas diffusion of phosphorus could be completely prevented by spin-coated silica-sol film placed on the rear side of the wafers coated prior to the diffusion process. A roughly 2% improvement in the conversion efficiency was observed when silica-sol was utilized during the phosphorus diffusion step. These results can suggest that the silica-sol material can be an attractive candidate for low-cost and easily applicable spin-coating barrier for any masking purpose involving phosphorus diffusion.

## Background

Crystalline silicon solar cells currently dominate the photovoltaic market, while ongoing research is mainly focused on increasing the conversion efficiency of the solar cells and the reduction of production costs. Junction formation is one of the most crucial steps during the solar cell fabrication process. Various methods are used to form homogenous phosphorus-diffused emitters for p-type silicon solar cells. These methods differ according to the technique used to deposit the phosphorus source onto the silicon surface, including deposition of phosphorus oxychloride (POCl_3_) [1,2], diluted orthophosphoric acid (H_3_PO_4_) by spray [3,4], sol-gel sources through spin-on deposition techniques [5], or by using the screen-printing technique [6]. However, the diffusion of phosphorus atoms to the rear surface cannot be avoided by either out-gas diffusion, regardless of the used phosphorus dopant source, or direct diffusion, such as in cases using POCl_3_. Therefore, it is necessary to mask the surfaces that one does not want to be diffused by phosphorus-doping atoms. SiO_2_ films are in use for many silicon device fabrications for either masking or passivation purposes. Conventionally, the deposition of SiO_2_ films using gas-phase deposition methods (atomic layer deposition [7,8], chemically catalyzed chemical vapor deposition [9], atmospheric-pressure chemical vapor deposition (APCVD) [10,11], low-pressure chemical vapor deposition (LPCVD) [10,11], and plasma-enhanced chemical vapor deposition (PECVD) [10]) have been performed. However, particle contamination and substrate surface damage come along as disadvantages [10] using CVD. Moreover, in some case, the use of dangerous silane gas is introduced [10,11]. Thermally growing oxide at high temperatures is another technique used to form oxide layers, either by dry oxidation using pure oxygen gas [12] or wet oxidation using oxygen/hydrogen steam [13]. Both are widely used in the fabrication of solar cells, especially for masking [2,14] and passivation purposes [15]. In order to pursue simple, high-performance and cost-effective production, the development of high-performance/low-cost materials and their adaption into the silicon solar cell fabrication process is crucial. This paper introduces a spin-coating silica-sol barrier material to protect against phosphorus diffusion. The silica sol barrier layer can be also applied by spray deposition or the screen printing method, should the proper modifications and improvements be made. However, such materials have yet to be investigated and reported sufficiently. It can be simply spun on the substrate surface prior to phosphorus diffusion and directly annealed simultaneously with phosphorus after the drying step. The performance evaluation of the silica-sol barrier material was carried out mainly in terms of sheet resistance measurements, secondary ion mass spectrometry (SIMS) analysis and scanning electron microscope (SEM) measurements. P-type CZ-Si solar cells were also fabricated both with and without using the silica-sol material during the phosphorus diffusion process.

## Methods

**Figure 1:**
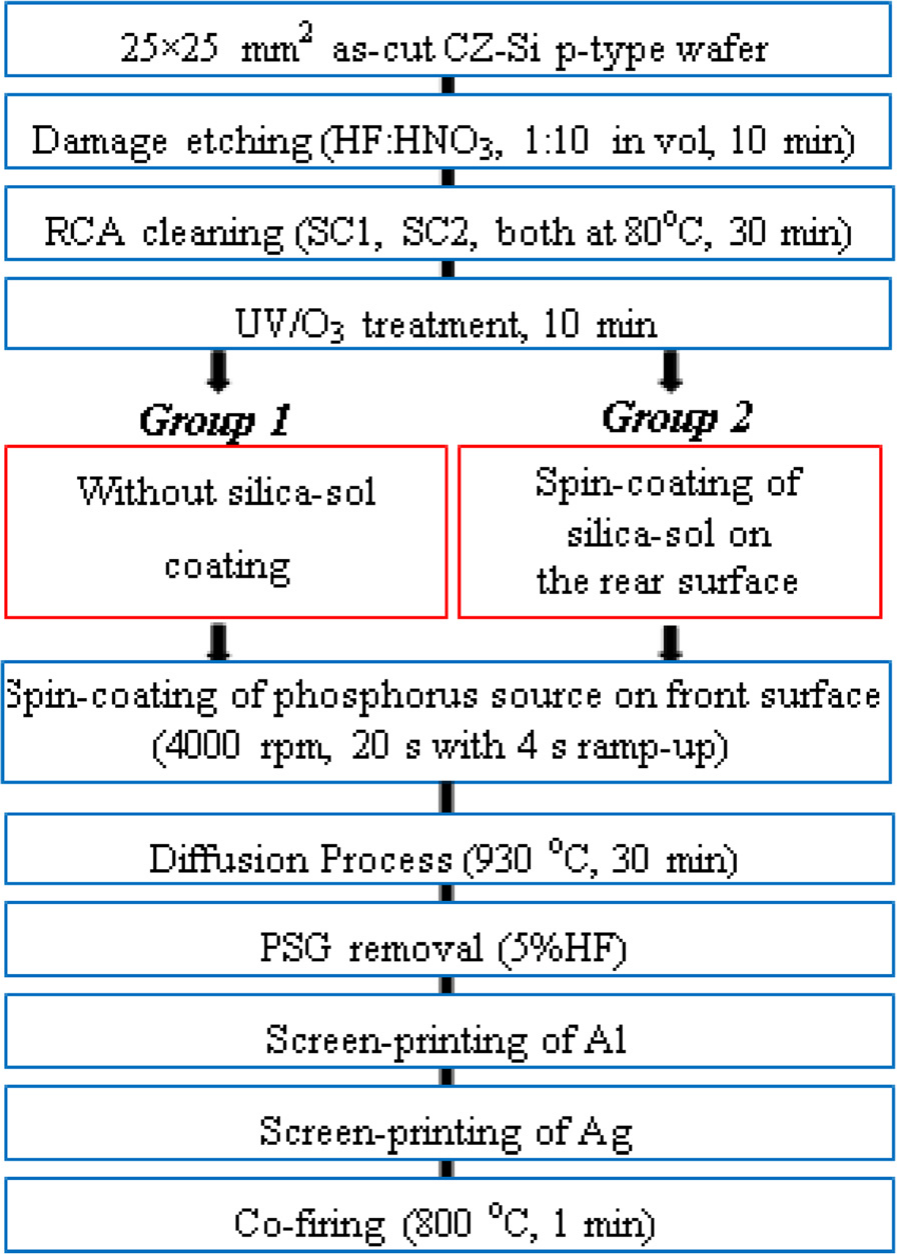
Experimental flow chart.

**Figure 2:**
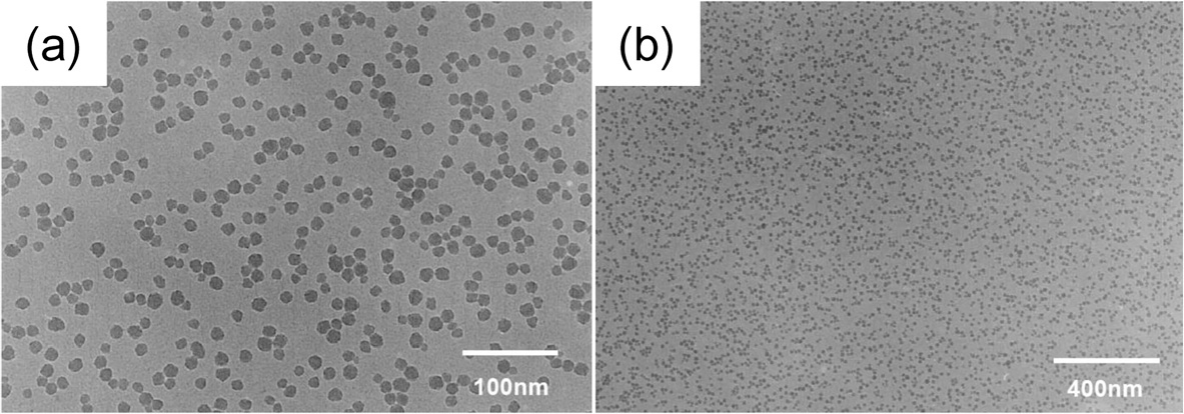
TEM images of silica solution: zoomed in (a) and out (b) views.

The experimental flow chart of the overall process is given in Figure 1. The 25 × 25-mm^2^-sized CZ-Si p-type wafers with thickness of 550 μm were used to evaluate silica-sol layer as a barrier for phosphorus diffusion. The silica-sol-based barrier (hereby referred to as ‘silica-sol’) was prepared by mixing silica nanoparticle dispersion (provided by Nissan Chemical Industries Ltd., Tokyo, Japan) with tetramethylammonium hydroxide (TMAH) by a ratio of 9:1 (in volume). The transmission electron microscopy (TEM) images of the silica solution material (the average diameter of silica particles is 22 nm (DLS) and 13 nm (BET); SiO_2_ concentration is 30.5%) are given in Figure 2, where the nanoparticles can be clearly seen with free dispersion. The experiments started with the removal of saw damage to the silicon wafers using an acidic etching, HF:HNO_3_ (1:10 in volume) for 10 min. Followed by the RCA cleaning procedure, standard cleaning-1 with NH_4_OH:H_2_O_2_:H_2_O (1:1:5 in volume) at 80°C for 30 min and standard cleaning-2 with HCl:H_2_O_2_:H_2_O (1:1:5 in volume) at 80°C for 30 min were performed to remove organic/inorganic contaminations. Before the spin coating of the silica-sol, UV/O_3_ treatment was performed to further clean the surface. After the cleaning process, the wafers were divided into two groups, to be diffused with only spin-coated phosphorus dopant source on the front side of the wafers (group 1) or to be diffused with spin-coated silica-sol on the rear side and spin-coated phosphorus dopant source on the front side (group 2). The spin coating of the silica-sol was carried out for 20 s with a ramp-up duration of 4 s. The spin speed was changed between 2,000 and 8,000 rpm. The phosphorus dopant agent was produced by mixing separately prepared H_3_PO_4_- and P_2_O_5_-based solutions (H_3_PO_4_ solution:P_2_O_5_ solution (1:10 in weight)). The H_3_PO_4_ solution included H_3_PO_4_:CH_3_COOCH_2_CH_3_:Si(OC_2_H_5_)_4_:(CH_3_)_2_CHOH (1:1.3:1.6:9.3 in volume). The P_2_O_5_ solution included P_2_O_5_:CH_3_COOCH_2_CH_3_:Si(OC_2_H_5_)_4_:(CH_3_)_2_CHOH (1:2:3:16 in weight). For the deposition of phosphorus-doping source, the spin speed was set to 4,000 rpm and coated for 20 s with ramp-up duration of 4 s. The spin-coated wafers were dried at 125°C for 3 min. The diffusion process was carried out at 930°C for 35 min. The silica-sol layer and phosphorus silica glass were easily removed using 5% diluted HF after the diffusion. The sheet resistances of the wafers were measured by a four-point-probe method (using Loresta-EP MCP-T36 tool by Mitsubishi Chemical Corp., Chiyoda, Tokyo, Japan) on both sides of the wafers after the diffusion process. The SIMS measurements were also carried out (using ADEPT-1010 (ULVAC-PHI, Chigasaki, Kanagawa, Japan) with etching rate of 0.668 nm/s, performed at NAIST). The uniformity of the coating was verified, and thicknesses were measured by SEM images (using JEOL-6510; JEOL Ltd., Tokyo, Japan). Once these silica-sol characteristics were recorded, silicon solar cells were fabricated and their electrical properties were compared. It is noteworthy to mention that all of the solar cells used in this experiment were fabricated without any passivation or antireflection coating layers and have non-textured surface in order to observe the effect of the silica-sol material more distinctly. Metallization of the front and rear contacts was performed by screen printing of Ag and Al, respectively. The printed metal pastes were dried at 125°C for around 5 min and then co-fired in an oven at 800°C for 1 min.

## Results and discussion

**Figure 3:**
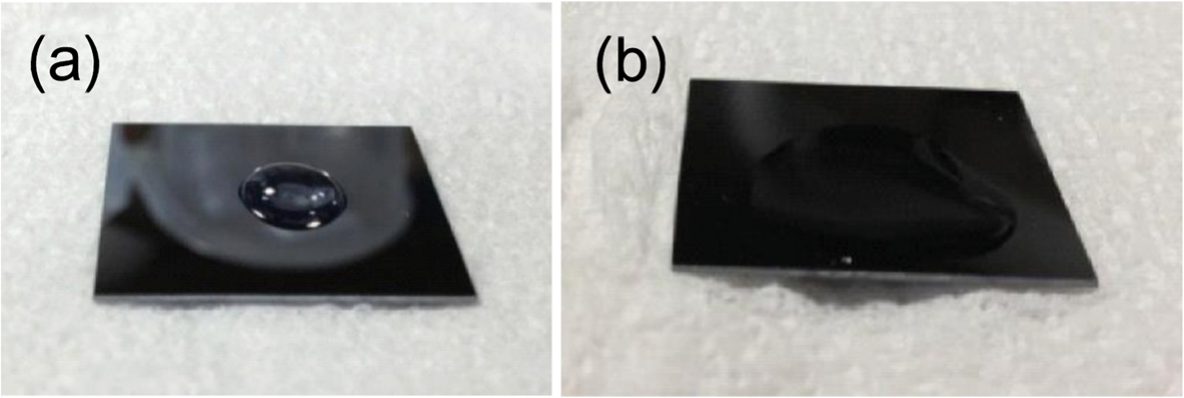
**Effect of ozone treatment on contacting quality of the solution on the surface. (a)** Dropping of the silica-sol on the wafer surface without applying UV/O_3_ treatment. **(b)** Dropping of silica-sol on the wafer surface after UV/O_3_ treatment.

Achieving a uniform coating is very crucial when one wishes to use a spin-coating material. UV/O_3_ treatment was applied in this work for further cleaning of the surface of the wafers and to assist homogenous distribution of the solutions. The effect of ozone treatment on the contacting quality of the solution to the surface is shown in Figure 3 using the actual images of the wafers. Without the application of the UV/O_3_ treatment, the silica-sol would not spread smoothly over the surface, accumulating with minimal contact with the wafer in a hydrophobic-like state (Figure 3a). However, the smooth spreading of the solution on the surface, which leads to a homogenous coating of the silica-sol through spin coating, could be achieved after the UV/O_3_ treatment (Figure 3b). This is due to the improved surface wettability, where the contaminant molecules are dissociated by the absorption of the short wavelength UV light and atomic oxygen/ozone is produced by the dissociation of O_2_, ending with the production of the volatile molecules [16,17].

**Figure 4:**
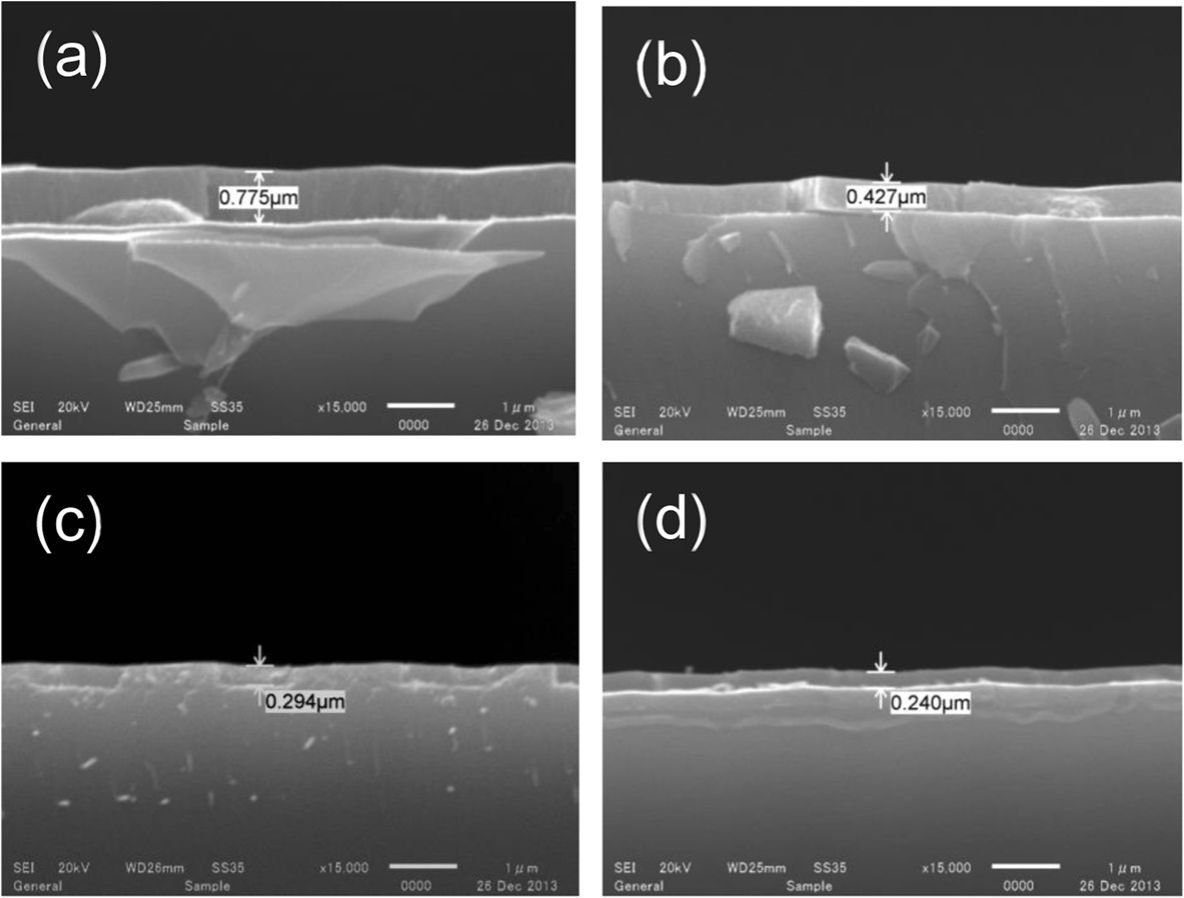
**Cross-sectional SEM images of spin-coated silica-sol films.** At 2,000 rpm **(a)**, 4000 rpm **(b)**, 6,000 rpm **(c)**, and 8,000 rpm **(d)**.

**Figure 5:**
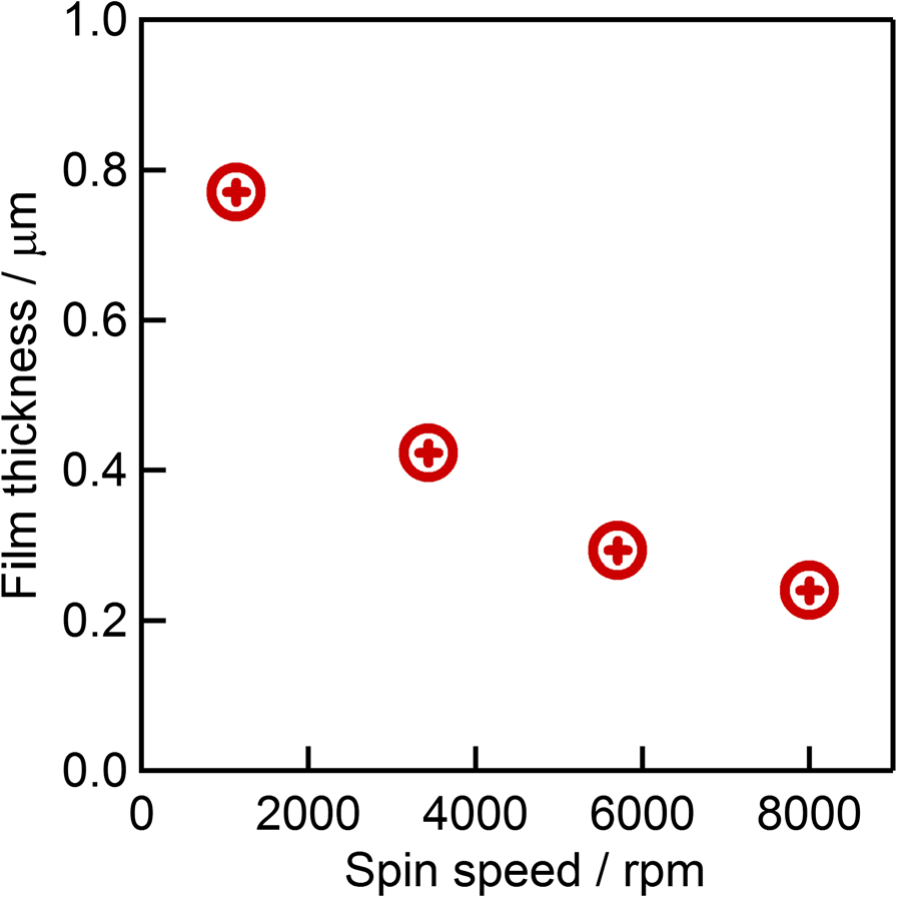
Effect on silica-sol film thickness due to the applied spin speed.

In order to investigate the optimum thickness of coated silica-sol, various spin speeds were applied (from 2,000 to 8,000 rpm) and the thicknesses were determined via the SEM images shown in Figure 4. A film thickness of 0.775 μm was observed after spin coating at 2,000 rpm. The coating thickness decreases with the increasing of spin speed; reaching roughly 0.3 and 0.240 μm at speeds of 6,000 and 8,000 rpm, respectively. The dependence of the film thickness to the applied spin speed is shown in Figure 5.

After the diffusion process, the phosphorus silica glass layer and silica-sol were removed using 5% HF treatment and the sheet resistances were measured on both sides. Table 1 shows the sheet resistance of group 1 and group 2 wafers. The sheet resistance of phosphorus-diffused sides was observed in a range of around 14 to 21 Ω/sq for all wafers. The sheet resistance measurements on the silica-sol-coated sides are shown as ‘over loading’ (out of the measurement range), indicating the blocking of any cross diffusions and/or out diffusions of phosphorus atoms on the rear side of the wafers. The barrier effect of the silica-sol could be confirmed regardless of the applied film thicknesses. However, the thickness of the silica-sol coating may affect the device performance. In order to investigate these possible effects, silicon solar cells were fabricated and electrical characteristics were compared and are discussed later on in this paper.

**Table 1:** Average sheet resistances of four samples measured on front and rear surfaces of the wafers

	**Spin speed for coating (rpm)**	**Front surface resistance (Ω/sq)**	**Rear surface resistance (Ω/sq)**
Group 1	Without silica-sol	21.4	350
Group 2	2,000	19.7	Over load
	4,000	14.5	Over load
	6,000	16.5	Over load
	8,000	20.0	Over load

**Figure 6:**
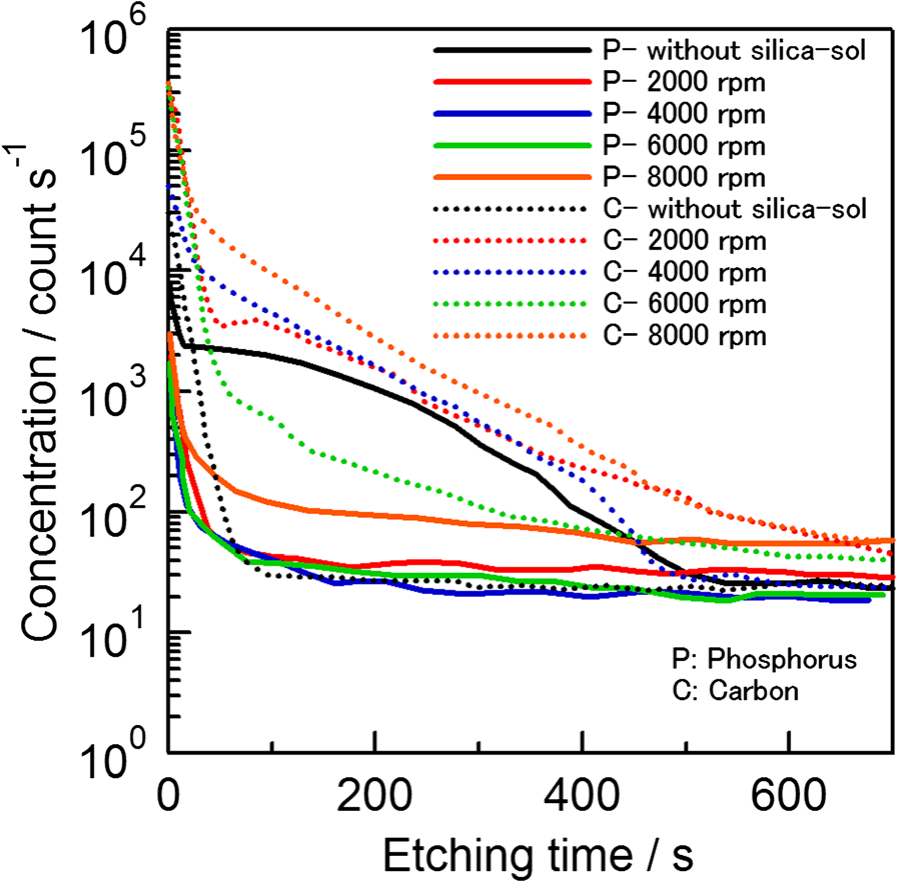
**Comparison of SIMS profiles of phosphorus and carbon atoms for group 1 and group 2 wafers.** This was done by measuring the rear side of the wafers after diffusion at 930°C.

The SIMS measurements were also carried out for further analysis. Figure 6 shows the SIMS profiles of phosphorus and carbon atoms, measured from the rear sides of the groups 1 and group 2 wafers after the diffusion at 930°C. For the wafer chosen from group 1, which had no silica-sol coating on the rear side during the diffusion, the phosphorus atoms diffused into the silicon mainly through the ambient. A peak intensity of 3 × 10^3^ counts/s phosphorus atoms was detected with a tail profile equivalent to a peak concentration of around 3 × 10^18^ cm^−3^, where the depth of the tail was around 0.8 μm (at 500 s in Figure 6). Since we were unable to have a standard reference for carbon concentration analysis available for the SIMS measurements, the y-axis of Figure 6 was set as the number of counts. The SIMS profiles of the silica-sol-coated wafers from group 2 were very similar and showed a total blocking of phosphorus atoms regardless of the applied spin speed or, in other words, the film thickness. The SIMS profiles of silica-sol-coated wafers from group 2, formed with spin speeds of 2,000, 4,000, and 6,000 rpm, were very similar. The slightly deeper-doped profile of the wafer coated with a spin speed of 8,000 rpm can be attributed to the comparably shallow film thickness. This barrier effect can be explained by the trapping of out-diffused phosphorus atoms in the silicon oxide and completely reacting to form a glass layer, leaving no more phosphorus atoms to diffuse through to the sufficiently thick silicon oxide. This is because, although the diffusion of phosphorus is very slow in an oxide layer, the phosphorus atoms actually react with the silicon oxide and form a mixed glass layer incorporating the phosphorus [18].

On the other hand, diffusion of carbon atoms was observed in all wafers of group 2 (Figure 6). The carbon concentration in group 1 wafers was observed to be the lowest level of all, with an intensity of around 2 × 10^4^ counts/s in a steadily decreasing attitude. However, higher carbon intensity with deeper profiles was observed for all group 2 wafers, which may come from the silica-sol paste. This diffusion of carbon has no degradation effect on the barrier film properties but needs to be investigated further. It is important to mention that although the silica-sol barrier paste in this report was investigated mainly for spin-on phosphate diffusions, some experiments were also tried out with unlimited sources like POCl_3_. However, only limited barrier effects could be observed for POCl_3_ source. Therefore, improvement of the silica-sol is in progress so that it may be applied to POCl_3_ diffusions as well.

**Figure 7:**
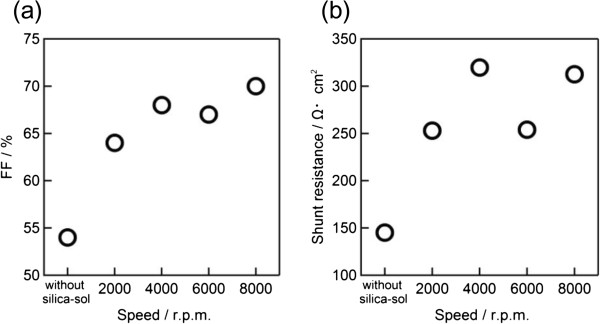
**Dependence of *FF *(a) and *shunt resistances *(b) of the fabricated cells to the rotation speed of the silica-sol.** The dependence formed on rear side of the wafers during the spin-coating phosphorus diffusion.

After the confirmation of the barrier effect of silica-sol (Figure 6), crystal silicon solar cells were fabricated by applying Ag and Al contact layers on the front and rear of the wafers without applying any passivation and antireflection layers. The electrical characteristics of the fabricated silicon solar cells are given in Table 2. *V*_*oc*_ and *J*_*sc*_ of the cells, from both groups 1 and 2 wafers, were observed to be around 20 mA/cm^2^ and 520 mV, respectively. However, the conversion efficiency and *FF* of the cells were increased by decreasing the applied silica-sol film thickness. The conversion efficiency of the cells that were fabricated without using silica-sol during the emitter formation (group 1) were around 6% with a *FF* of 54%. For those with the silica-sol film on their rear side (group 2), the cell efficiency rose more than 1%, reaching up to 8.3% for those cells with thinner silica-sol coating (0.24 μm) with a *FF* of 70%. The comparisons of *FF vs. shunt resistances* were provided in relation to the rotation speed of the silica sol, in Figure 7a,b, respectively. Although shunt resistances were not too high, they show a good agreement with the *FF* of the cells; *FF* increased with the increase of shunt resistance. It should be considered that the silica sol barrier may not cover the edge of the wafers and, as such, may not totally avoid the shunting but does prevent the rear-side phosphorous diffusion thanks to a full and even coating. These results can be attributed to the blocking of phosphate out-gas diffusion, which may also lead to a uniform back-surface field. Since the evaluation of silica-sol film and its effects on cell properties were the main target of this work, a simple solar cell fabrication process was applied with only emitter and contact formations. Therefore, the generally poor cell results are due to the lack of any antireflective coating, non-textured surfaces, and the low quality of the silicon bulk. It can be concluded that the silica-sol-based barrier material introduced in this work can be an attractive material to block phosphorus diffusion by using a simple and cost-effective spin-coating method. It can be adapted into the solar cell fabrication process as a barrier for phosphorus diffusion instead of other masking methods, e.g., thermal oxidation and vacuum-process oxide deposition.

**Table 2:** Electrical characteristics of fabricated solar cells with/without using silica-sol on rear side during phosphorus diffusion

**Solar cell parameters**	**Group 1**	**Group 2 applied spin speed (resulted film thickness)**			
	**Without silica-sol**	**2,000 rpm (0.77 μm)**	**4,000 rpm (0.43 μm)**	**6,000 rpm (0.30 μm)**	**8,000 rpm (0.24 μm)**
***J***_**sc **_**(mA/cm**^**2**^**)**	22.2	22.1	20.7	21.7	22.2
***V***_**oc **_**(mV)**	515	521	522	522	525
***FF *** **(%)**	54	64	68	68	70
***Eff *** **(%)**	6.19	7.37	7.32	7.62	8.12

All values are average values of three cells. Eff, efficiency.

## Conclusion

A spin-coating silica-sol material was introduced as a promising barrier material for phosphorus diffusion. The out diffusion of phosphorus could be completely prevented by using silica-sol-based film prepared using mixture of silica-sol dispersion with TMAH (9:1 in volume). After the evaluation of the material, the silicon solar cells were fabricated both with and without using the silica-sol. Conversion efficiency improvement was observed up to around 2% when utilizing silica-sol during the phosphorus diffusion step. These results can lead to the use of simple, cost-effective and high-performance silica-sol material in the silicon solar cell fabrication process. It is clear that the thermal budget of this process is lower than those of the usual techniques. The material is also cheap to produce, with the actual chemical (silica-sol) price shifting significantly according to the production volume, which will be considered in the future production stage.

Actually, it should be worth comparing the effect of the silica-sol layer with samples using currently used diffusion barrier layers (SiO_x_ or SiN_x_) than with samples using no barrier at all. However, applying a non-solution-based barrier layer requires adding more steps to the process, the use of expensive equipment, etc. In any event, the development of a solution-based diffusion barrier material, as well as its application through spin coating, was the goal of this work. Indeed, the spin-coating process is used widely, mainly at laboratory scales, for fabricating thin films. We aimed to coat, and evaluate, silica sol on flat surfaces by spin coating first due to the inherent difficulties when trying to apply it to a textured surface. In the future steps, spray deposition or screen printing methods may become possible, but the necessary modifications and improvements to the paste are still under development at this time.

## Competing interests

The authors declare that they have no competing interests.

## Authors’ contributions

AU supervised the research and drafted the manuscript. KF carried out the devise fabrication in this study. YK and KT served the SiO_2_ nanocolloid. SY, YJ, YI and YU measured SIMS. SI supervised the research, organizing the laboratory for this work and finalized the manuscript. All authors read and approved the final manuscript.

## Authors’ information

AU is a postdoc researcher in University of Hyogo. KF was a Master course student in University of Hyogo. YK and KT are researchers in Nissan Chemical Industry Co. Ltd. (Japan). SY and YJ are students in NAIST (Japan). YI is an Associate Professor in NAIST (Japan). YU is a Professor in NAIST (Japan). SI is an Associate Professor in University of Hyogo (Japan).
